# Public perception and performance of different sampling approaches for the diagnosis of COVID‐19

**DOI:** 10.1111/irv.12834

**Published:** 2021-01-04

**Authors:** Ngai Yung Tsang, Hau Chi So, Hiu Fai Ho, Gabriel M. Leung, Dennis K. M. Ip

**Affiliations:** ^1^ WHO Collaborating Centre for Infectious Disease Epidemiology and Control School of Public Health Li Ka Shing Faculty of Medicine The University of Hong Kong Hong Kong SAR China; ^2^ Accident & Emergency Department Queen Elizabeth Hospital Hong Kong SAR China

To the Editor,

For the diagnosis of SARS‐CoV‐2 to inform timely and appropriate clinical management in an inpatient setting, a number of alternative respiratory specimens, including pooled nasal and throat (N&T) swabs, nasal swabs, throat swabs and saliva or sputum, have been explored.^1^, ^2^ On the other hand, self‐collection of specimens was being increasingly advocated to cater to the expanding testing needs in community settings, for the theoretical benefit of saved manpower requirement and risk minimization for healthcare workers. Comparing to nasopharyngeal swab as the standard sample type accepted for diagnosing COVID‐19,^3^ some studies reported saliva sample as an non‐invasive specimen with good diagnostic performance,^4^, ^5^ while others were either having conflicting results^6^ or had cautioned the potential of a reduced sensitivity when used in outpatient setting with a low disease prevalence.^7^ The overall relative diagnostic accuracy of different self‐collected specimens, besides remained to be inconclusive, may also be highly dependent on patient's acceptance, confidence and understanding of the required self‐collection process.

Here, we present our study in a cohort of mild community cases in Hong Kong, comparing reverse transcription polymerase chain reaction (RT‐PCR) results from early morning deep throat saliva with pooled N&T swabs, with the finding of a good testing performance. As self‐collection samples were being increasingly advocated for the potential benefit of reduced manpower requirement and risk of exposure,^8^ our results highlighted the importance of taking patients’ differential acceptance and confidence for performing self‐collection of different samples into consideration when expanding diagnostic testing in the community.

We conducted a cross‐sectional study to assess the relative performance and perception of different sampling approaches among adult symptomatic patients presenting to the Accident & Emergency Department of a regional public acute hospital during the heightened period of COVID‐19 epidemic (30/3/2020‐28/7/2020) in Hong Kong. Patients classified as “Tier 4” under the surveillance system were recruited, specifically including clinically stable outpatients presenting with COVID‐19–associated symptoms and having no definitive travel or contact history necessitating quarantine, nor being clinically severe to the extent of indicating for hospitalization.

In parallel to the self‐collected saliva sample prescribed by attending physicians, an additional pooled N&T swabs collected by our nurses from each consented patient were tested for RT‐qPCR assay targeting the N gene of SARS‐CoV‐2. N&T swabs were collected by rotating a sterile plain swab (CLASSIQSwabs™; Copan) into the anterior nasal cavity and another swab in the tonsillar fossae. Anterior nasal swab is considered less invasive and is widely accepted in our previous community studies on respiratory virus.^9^ After sample collection, participants were interviewed for their perception basing on their experience with the two sampling approaches. The study protocol was approved by Research Ethics Committees of the HKU (UW 20‐196) and Hospital Authority (KC/KE‐20‐0067/ER‐3).

## 1 PUBLIC PERCEPTION

**FIGURE 1:**
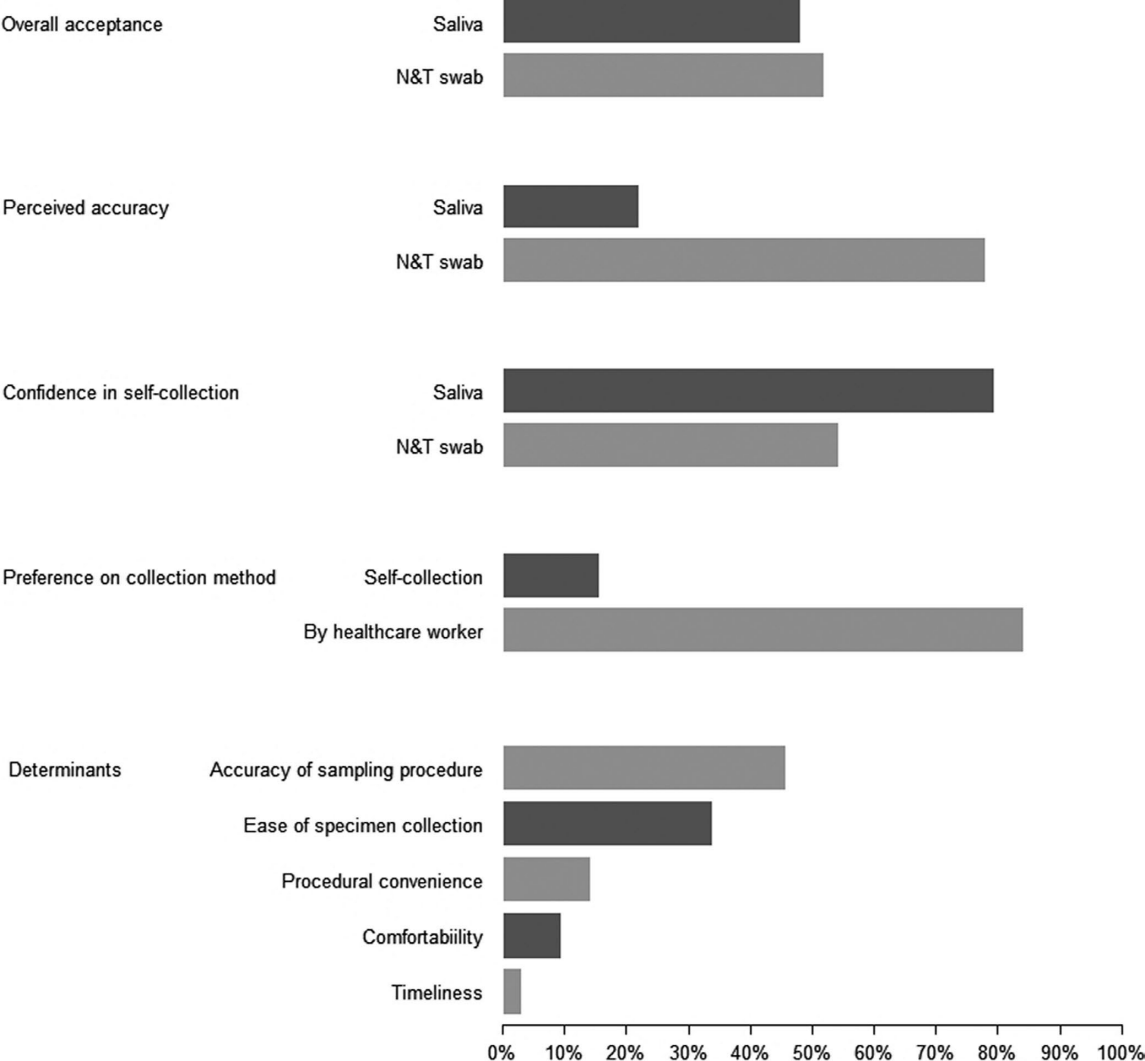
Public preference on different sampling approaches for diagnosis of community COVID‐19 infection

A total of 127 participants completed the survey. Although acceptance of the two different sampling approaches appeared grossly comparable (52% vs 48%, *P* = .65), the majority (78%) of respondents actually perceived pooled N&T swab to be a more accurate sampling approach for diagnosing COVID‐19 in a primary care setting (*P* < .01) (Figure 1). A much higher confidence was reported for the accurate self‐collection of saliva than pooled N&T swab sample (80% vs 54%, respectively, *P* < .01), with most individuals (84%) preferring the swabs to be collected by a healthcare worker instead (*P* < .01) (Figure 1). Common concerns affecting the acceptability of different sampling approaches included accuracy of sampling procedure (46%), followed by ease of specimen collection (34%), procedural convenience (14%), comfortability (9%) and timeliness (3%) (Figure 1).

## 2 DIAGNOSTIC PERFORMANCE

A total of 402 pairs of saliva and pooled N&T swabs were collected, 215/402 (53.5%) were female and with a median age of 38 years (range: 18‐86). Eight patients (1.9%) were confirmed to have COVID‐19 infection by RT‐PCR. Six of them were positive by both saliva and pooled swabs samples, with each of the remaining 2 positives by only saliva (1) or N&T swabs (1). Using N&T swabs as the reference standard, saliva samples were having a sensitivity and specificity of 85.7% (95% CI 42.1%‐99.6%) and 99.7% (95% CI 98.6%‐100.0%), and a positive and negative predictive values of 85.7% (95% CI 42.1%‐99.6%) and 99.7% (95% CI 98.6%‐100.0%), respectively. There was a high concordance (99.5%, κ = 0.86, 95% CI 0.66‐1.00, *P* < .01) between saliva and N&T swabs.

In conclusion, our results from a cohort of mild community cases in Hong Kong, with a low disease prevalence, did not demonstrate a similar reduction in the diagnostic performance of saliva sample comparing to N&T swabs as reported in other similar community setting.^7^ The comparable diagnostic performance for both sampling approaches supported their suitability for being used in community screening programme where the disease prevalence may be low, especially when there is a shortage of personal protective equipment during the pandemic. On the other hand, although self‐collection of both specimens is generally believed to be a straightforward procedure, our results revealed that people can have very different acceptance and confidence for performing self‐collection of different samples. While the benefit of manpower saving and exposure reduction offered by self‐collected saliva sample is readily appreciable, the much lower perceived capability of self‐collection and the vast majority of people preferring its collection by healthcare workers highlighted the potential caution for the case of N&T swabs. With the rapidly evolving epidemic situation of COVID‐19 in different countries, an increasing need for the accurate and efficient popularization of testing is expected. Further work is needed to better assess patients’ understanding and suitability of different sampling procedures in different settings, and to inform evidence‐based policy decision and programme implementation. This issue remains largely unexplored currently in the relevant literature.^10^

## CONFLICT OF INTEREST

All authors declare that there are no conflicts of interests.

## AUTHOR CONTRIBUTIONS

**Ngai Yung Tsang:** Formal analysis (lead); Visualization (lead); Writing‐original draft (lead). **Hau Chi So:** Formal analysis (equal); Project administration (lead); Writing‐review & editing (equal). **Hiu Fai Ho:** Writing‐review & editing (equal). **Gabriel M. Leung:** Writing‐review & editing (equal). **Dennis KM Ip:** Conceptualization (lead); Methodology (lead); Writing‐review & editing (lead).

## DATA AVAILABILITY STATEMENT

The data that support the findings of this study are available from the corresponding author, DKMI, upon reasonable request.
